# Saliva sampling in global clinical studies: the impact of low sampling volume on performance of DNA in downstream genotyping experiments

**DOI:** 10.1186/1755-8794-6-20

**Published:** 2013-06-10

**Authors:** David J Pulford, Michael Mosteller, J David Briley, Kelley W Johansson, Anita J Nelsen

**Affiliations:** 1 GlaxoSmithKline, Research & Development, Medicines Research Centre, Gunnels Wood Road, Stevenage, Herts SG1 2NY, UK; 2 GlaxoSmithKline, Research & Development, Five Moore Drive, Research Triangle Park, NC 27709, USA

**Keywords:** Global study, Volume of saliva collection, DNA characteristics, Genotyping performance

## Abstract

**Background:**

The collection of viable DNA samples is an essential element of any genetics research programme. Biological samples for DNA purification are now routinely collected in many studies with a variety of sampling methods available. Initial observation in this study suggested a reduced genotyping success rate of some saliva derived DNA samples when compared to blood derived DNA samples prompting further investigation.

**Methods:**

Genotyping success rate was investigated to assess the suitability of using saliva samples in future safety and efficacy pharmacogenetics experiments. The Oragene® OG-300 DNA Self-Collection kit was used to collect and extract DNA from saliva from 1468 subjects enrolled in global clinical studies. Statistical analysis evaluated the impact of saliva sample volume of collection on the quality, yield, concentration and performance of saliva DNA in genotyping assays.

**Results:**

Across 13 global clinical studies that utilized the Oragene® OG-300 DNA Self-Collection kit there was variability in the volume of saliva sample collection with ~31% of participants providing 0.5 mL of saliva, rather than the recommended 2 mL. While the majority of saliva DNA samples provided high quality genotype data, collection of 0.5 mL volumes of saliva contributed to DNA samples being significantly less likely to pass genotyping quality control standards. Assessment of DNA sample characteristics that may influence genotyping outcomes indicated that saliva sample volume, DNA purity and turbidity were independently associated with sample genotype pass rate, but that saliva collection volume had the greatest effect.

**Conclusion:**

When employing saliva sampling to obtain DNA, it is important to encourage all study participants to provide sufficient sample to minimize potential loss of data in downstream genotyping experiments.

## Background

The increasing demand for personalized medicine has seen an increase in the biobanking of human biological specimens, and in particular genomic DNA samples, from individual participants enrolled in clinical studies. The FDA has recently emphasized the importance of prospective DNA sample collection as a prerequisite to the successful use of genetic information in drug development [1]. Indeed, for many pharmaceutical companies the collection of DNA samples is now a routine part of clinical trials [2]. Many clinical study teams embark on prospective DNA sample collection that may allow downstream molecular analysis aimed at characterizing disease and response to therapy. The collection of high quality genomic DNA samples is clearly an essential element of genetics research.

Once collected, samples are transported, frequently internationally, for pre-analytical processing at a limited number of central laboratories to extract DNA. The central laboratory employs high throughput standardized protocols to extract DNA prior to long term storage and genetic analysis. Thus, the processes of sample collection, DNA extraction, storage and use are carried out according to standard operating procedures. Each step is critical to ensure the delivery of high quality DNA.

Study participants provide a biological sample, routinely a whole blood sample, although non-invasive sample collection methods may be employed. Alternative collection methods that may include buccal swabs, mouthwash, cytobrush and blood spot filter card, while effective, are not without problems and may only collect small quantities of tissue and hence yield low amounts of DNA [3,4]. Non-invasive saliva sampling is often used where blood sampling is not desirable. Commercially available collection kits can provide yields of DNA from saliva that are comparable to the same volume of blood and of sufficient quality for use on many custom genotyping platforms [4]. Indeed, saliva DNA is reported to generate comparable results to blood DNA in both Taqman and genome wide genotyping assays [5] as long as the mean amplifiable percentage of human DNA is sufficient [6]. However, commercially available collection kits generally require study subjects to provide only a small volume of saliva (between 1 and 2 mL), in contrast to many studies using blood sampling where perhaps 6–10 mL of blood is collected [4,5]. Thus, when saliva sampling is used it is reasonable to expect lower yields of DNA. Hence, it is important that the correct volume of saliva is collected to avoid impact on potential downstream processes that could result in DNA samples being at sub-optimal yield or concentrations, below that routinely required for current genotyping platforms, resulting in higher failure rates during genotyping.

An initial analysis of genotyping data from three independent global clinical trials indicated that genotype results from saliva or blood derived DNA were comparable, in agreement with previous reports [5], but that saliva DNA samples were less likely to pass genotyping quality control standards. Specifically, results from the 3 trials suggested that DNA samples where the initial volume of saliva collection was 0.5 mL were most likely to fail genotyping quality control standards. In order to further investigate the effect of saliva sample volume on the quality of resulting DNA, saliva samples and extracted DNA from over 1400 subjects who were participants in 15 GlaxoSmithKline (GSK) clinical studies were evaluated.

## Methods

### Sample procurement, processing and storage

The present analysis was based on samples collected in 15 global GSK sponsored clinical studies, 14 of which involved subjects with respiratory disease. The remaining study was a comparative study in healthy and type 2 diabetes subjects. Studies were conducted across 282 clinical sites in 13 different countries from Europe, North America, South America, Africa and Asia. All study protocols described the collection of a DNA sample for genetic research, including sample quality control (QC) analysis. All study subjects or where appropriate their legally authorized representative provided written informed consent for genetics research prior to the collection of a human biological sample. All studies were conducted in accordance with the declaration of Helsinki and International Conference on Harmonization Good Clinical Practice (ICH-GCP) requirements. All protocols and informed consent forms were reviewed and approved by national, regional and Institutional Review Boards/Independent Ethics Committees (IRB/EC) according to local guidelines and details are available in the Additional file 1.

Study participants provided either a saliva sample collected using the Oragene® OG-300 non-invasive saliva sampling kit (DNA Genotek) or 10 mL whole blood sample collected into an EDTA vacutainer (Becton Dickinson). Samples were shipped ambient on day of collection. Germ-line genomic DNA was extracted by Quest Diagnostics on the Autopure LS utilizing Gentra Puregene® (Qiagen). Automated sample preparation incorporated a step to calculate the volume of sample collection based on sample weight and solution density of 1 g/mL. DNA pellets were re-suspended in TE buffer (10 mM Tris–HCl, 10 mM EDTA, pH8.0) to a final volume of 500 μl (saliva DNA) or 900 μl (blood DNA). DNA sample yield, concentration and A_260/280_, A_260/230,_ and A_320_ data was provided by Quest Diagnostics or by Gentris (Morrisville, NC). UV absorbance values were determined on a NanoDrop ND-1000 or NanoDrop-2000 Spectrophotometer (Thermo Scientific). DNA samples were stored in secure facilities at −20°C. Distributions for saliva DNA concentration, yield and turbidity were noticeably skewed and a natural logarithm transformation was applied to the values used in subsequent analysis.

### Genotyping

The impact of sampling method was evaluated by comparing genotyping experimental outcomes for DNA derived from either blood or saliva samples. Initially a genome wide association study (GWAS) using the Illumina Human OmniQuad BeadChip was performed by Expression Analysis (Durham, NC) using DNA derived from 551 saliva samples and 352 blood samples, respectively. Additionally, Fluorescent Resonance Energy Transfer KASPar (FRET) genotyping assays [7] were conducted for 77 single nucleotide polymorphisms (SNPs) on the same sample cohort. For KASPar assays, there were 43 overlapping SNPs with the Illumina Human OmniQuad BeadChip. Comparison of overlapping data for blood and saliva derived DNA, were both highly concordant producing a >99.5% concordance rate between the 2 genotyping platforms [Additional file 2: Table S1].

Further experiments focused on DNA derived from saliva collected from consenting subjects who were enrolled in any one of 13 global clinical studies. Genotyping was performed on DNA derived from 1468 subject saliva samples utilizing FRET KASPar assays for 220 SNPs. In this experiment, for 218 subjects, duplicate DNA samples were included to yield a total number of 1686 DNA samples. Additionally, subject overlap from the first cohort was included to confirm findings seen within the first experiment. All FRET genotyping assays were performed at Kbiosciences (Hoddesdon, UK).

During genotype quality control (QC), a sample was considered to have passed genotyping if 95% of markers tested successfully returned a genotype. Where samples passed genotyping QC, comparisons of genotype data generated from duplicate samples demonstrated a high degree of concordance. Among 41,586 comparisons there were only 26 discrepancies (concordance rate 99.94%, 95% confidence interval (CI): [99.91%, 99.96%]). The distribution of minor allele frequencies was fairly uniform over the range of 0 to 0.5.

### Statistical analysis

A likelihood ratio chi-square statistic was used to test for association between categorical variables. Simple linear regression or one-way analysis of variance models were used to assess associations with continuous variables. Logistic regression was used to evaluate the effects of multiple variables on the binary variable that indicated a sample’s genotyping QC status (that is, pass/fail). All other comparisons were descriptive.

## Results

### Comparison of blood and saliva DNA genotyping performance

**Figure 1:**
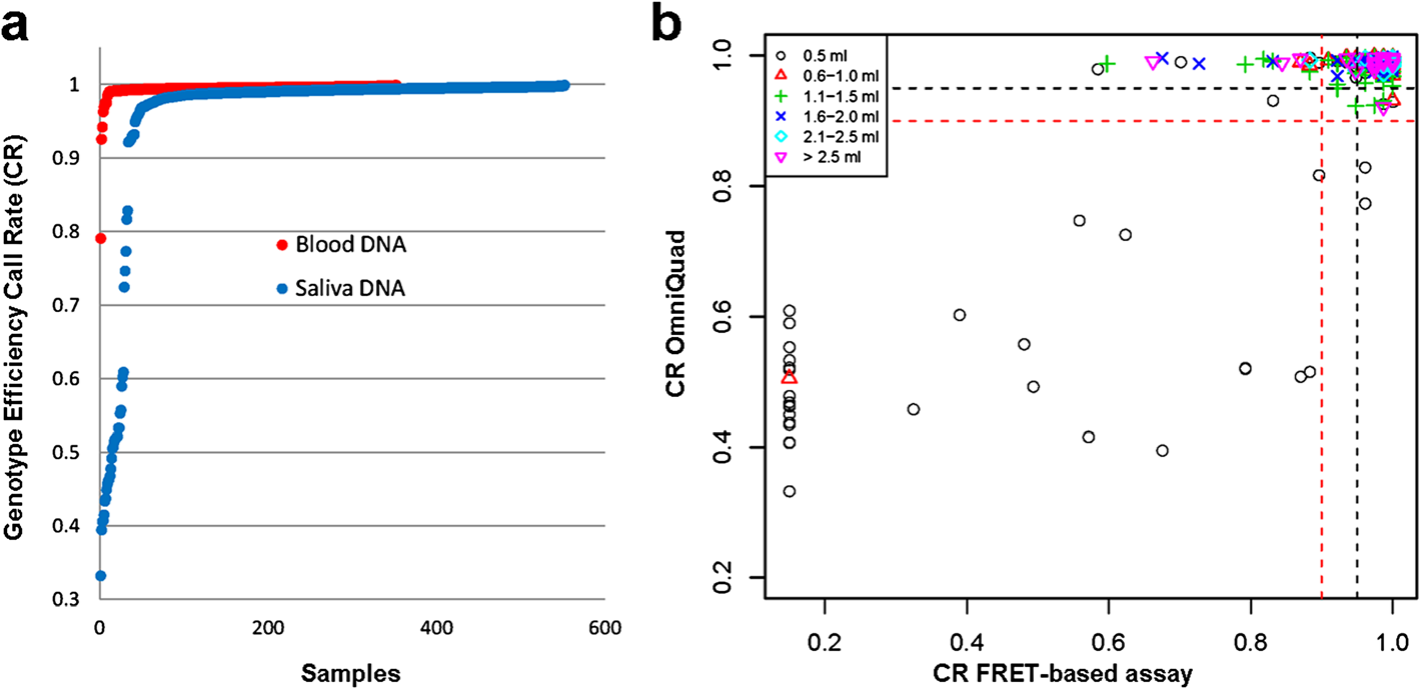
**PGx sampling method and genotype efficiency call rate.** (**a**) Call rate distribution by sample type: Single nucleotide polymorphism data from saliva derived or blood derived DNA was generated using the Illumina Human OmniQuad BeadChip. One hundred ninety-eight (36%) of 551 saliva samples tested failed genotyping according to manufacturer defined QC standards compared to 11 of the 352 (3%) blood DNA samples (p < 0.0005). Similarly saliva DNA samples were more likely than blood DNA samples to fail genotyping QC criteria on the FRET-based KASPar platform. Eighty-two of 447 saliva DNA samples (18.3%) tested failed genotyping QC standards compared to 21 of 331 (6.3%) blood DNA samples (p < 0.001; not shown). (**b**) Genotype call rate and saliva collection volume: Saliva DNA samples where the collection volume was 0.5 mL were more likely to provide low quality genotype data on both OmniQuad and FRET-based genotyping platforms.

We first evaluated the impact of sampling method on genotyping experimental outcomes. Single nucleotide polymorphism (SNP) data from 551 saliva and 352 blood DNA samples, respectively, was generated using the Illumina Human OmniQuad BeadChip and a FRET-based KASPar platform. Analysis of these data showed that saliva DNA samples were less likely to pass genotyping quality control standards. Analysis of genotype data for 551 saliva DNA samples on both genotyping platforms indicated that samples where the initial volume of saliva collection was 0.5 mL performed particularly poorly [Figure 1].

### Global saliva sample acquisition

Given initial evidence that low volume saliva samples were more likely to fail to genotype, we investigated compliance with recommended collection practices across 13 global clinical studies. The manufacturer’s recommended collection volume for saliva using the Oragene® OG-300 non-invasive saliva sampling kit is 2 mL. Non-invasive saliva samples were available from 1468 study subjects that participated in 13 clinical studies and also consented to genetics research. However, among the 13 clinical studies, compliance with the collection protocol was inconsistent. The average saliva volume collected was 1.15 mL but ranged from 0.5 mL to 3.2 mL, although the central laboratory reported that saliva sample volume may have been less than 0.5 mL in some cases. A total of 456 samples (~31%) of the 1468 that were collected had a reported volume of 0.5 mL. At the other end of the volume range, 37 (2.5%) had volumes greater than 2.4 mL. A review of the 13 clinical studies indicated that the frequency of 0.5 mL saliva samples differed significantly among studies (P < 0.0001, data not shown), possibly indicating inconsistent collection practices among the study sites.

Since high throughput DNA extraction protocols require that DNA is re-suspended in a standard volume of TE, low volume saliva sample may result in DNA samples that are below the optimal DNA concentration required for some genotyping platforms.

The average DNA yield was 93 μg and ranged from 12 μg to 435 μg. Not surprisingly, there was a clear relationship between the category of saliva sample volume (0.5 mL versus >0.5 mL) and the quantity of DNA extracted. For samples with volumes greater than 0.5 mL, the average DNA yield was 99 μg, whereas for 0.5 mL samples it was 81 μg. The standard volume of TE that a DNA pellet is re-suspended in is 500 μl. Our analysis demonstrated that the mean volume of re-suspended DNA was 490 μl (95% CI: [487, 493]) and that the mean final DNA concentration of samples was 188 ng/μl (95% CI: [181, 196]), but ranged from 21 ng/μl to 826 ng/μl. Of the saliva samples collected, 12% yielded DNA samples at concentrations below 50 ng/μl, the concentration typically requested by many genotyping vendors. Approximately 38% of these low yielding saliva samples had a reported collection volume of 0.5 mL. In contrast, of the saliva samples that yielded DNA samples at a concentration ≥50 ng/μl, approximately 30% had a reported collection volume of 0.5 mL (P = 0.03), suggesting that low volume saliva samples were more likely to yield DNA samples at a concentration below 50 ng/μl.

### Volume of saliva collection and genotyping success

**Figure 2:**
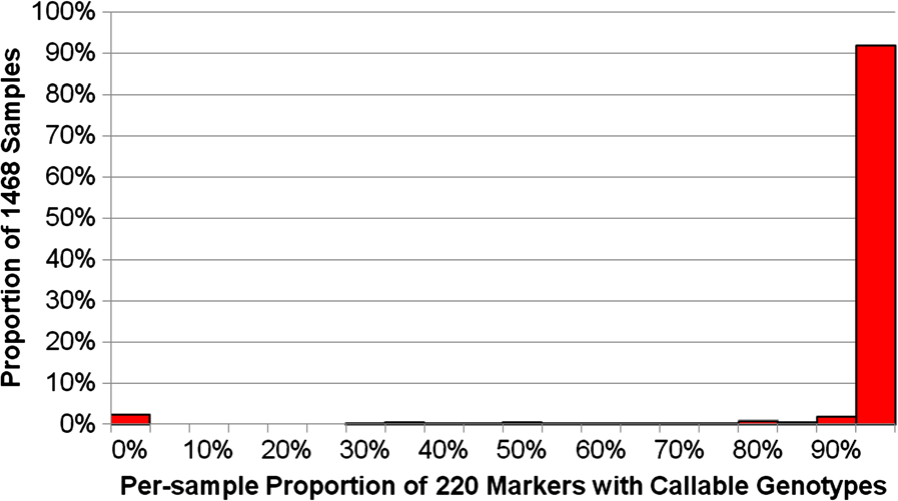
Distribution of per-sample call rates.

Genetic data from 13 clinical trials were evaluated to assess the impact of saliva sample volume on genotyping experimental outcomes on the FRET-based KASPar genotyping platform. For each sample, the proportion of the 220 markers that yielded callable genotypes was calculated. Figure 2 shows the distribution of these proportions among the 1468 samples tested. Notably, 34 samples failed to generate genotypes for any of the markers tested. In total, 122 (8.3%) samples yielded genotype results for fewer than 95% of the markers tested. However, across all samples the median call rate was 99.5% and 91.7% of samples yielded genotype results for at least 95% of the markers, demonstrating that DNA extracted from saliva samples, in general, provides robust genotype data.

There was a strong association between sample volume and successful genotyping outcome. For DNA samples where the saliva collection volume was >0.5 mL the pass rate was 96.7%, whereas for DNA samples where the collection volume was 0.5 mL, the pass rate was substantially lower at 80.5% (P < 0.0001; Table 1). Thus, saliva DNA, where the initial volume of saliva collection was 0.5 mL, performed less well for the genotyping platform utilized. These results suggest that sample collection volume is an important factor that contributes to successful genotyping experimental outcomes.

**Table 1:** Sample status by saliva sample volume category

**Sample status**	**Saliva sample volume**					
	**>0.5 mL**		**0.5 mL**		**Total**	
	**N**	**%**	**N**	**%**	**N**	**%**
Failed	33	3.3	89	19.5	122	8.3
Passed	979	96.7	367	80.5	1,346	91.7
Total	1,012	100.0	456	100.0	1,468	100.0

**Figure 3:**
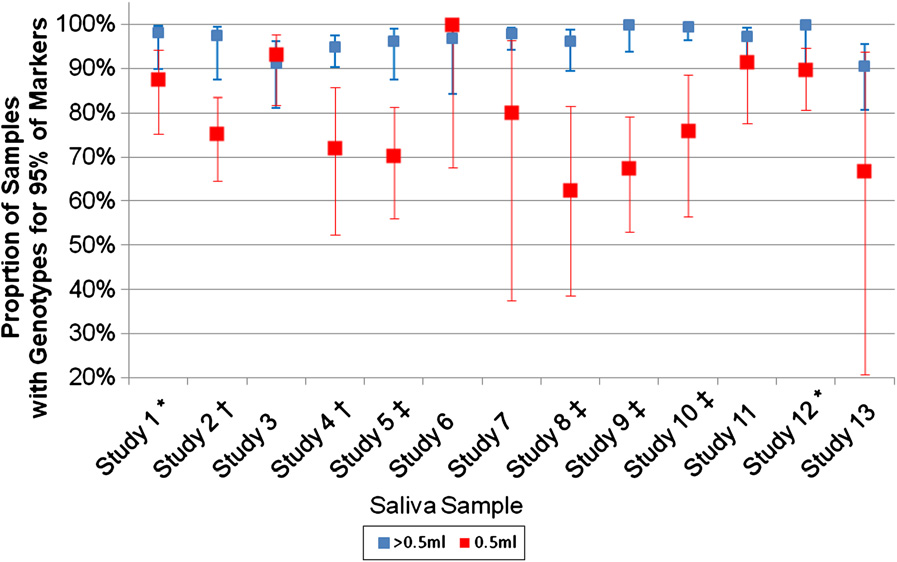
**Sample pass rates by sample volume and study, with 95% CIs.** Symbols following each study indicate the results of a statistical test that compared the pass rates of samples with a volume of 0.5 mL versus samples with a volume greater than 0. 5 mL (no symbol: P > 0.05; *: P < 0.05; †: P < 0.01; ‡: P < 0.001).

Somewhat unexpectedly, the genotyping performance of low volume saliva samples was not consistent across the 13 clinical studies. For low volume samples (0.5 mL saliva) sample pass rate ranged from 63%-100%. Indeed, a likelihood ratio test to assess sample pass rate for low volume samples indicated that sample pass rate for low volume samples was significantly different between studies (P = 0.002) suggesting there is an interaction between sample volume and study with respect to sample pass rate (Figure 3). For eight of the studies, there was evidence for the pass rate in >0.5 mL samples being greater than the pass rate in 0.5 mL samples (Fisher’s Exact Test P < 0.05) for the remaining five studies the pass rates in the two volume categories were not statistically different. Indeed, in two studies the sample pass rate was slightly higher in the 0.5 mL group compared to the >0.5 mL group. Thus, while in general low volume saliva samples were more likely to result in lower sample pass rates, this pattern was not consistently observed, suggesting that practices unique to the clinical studies during sample acquisition and processing might also have influenced the outcome. Since the vast majority of subjects (>97%) who provided saliva samples were enrolled into respiratory studies, analysis by disease would not have been meaningful. Interestingly, a borderline significant association (P = 0.058) was observed between age and saliva volume, suggesting that older subjects may have been more likely to provide a 0.5 mL sample, consistent with reports that xerostomia (dry mouth) prevalence increases with age [8].

### Saliva DNA sample purity and turbidity contribute to genotyping success

Additional variables that may influence DNA sample genotyping pass rate were evaluated across all samples. Analysis of UV absorbance (A_260_/A_280_ and A_260_/A_230_) ratios across all samples established that greater than 99% of samples were of high purity and that there was little evidence of contaminating organic compounds or chaotropic salts that may have been carried over through the DNA extraction process and contributed to poor genotyping outcomes.

Amongst the variables that were individually tested for association with sample pass/fail status, only DNA purity and turbidity were associated at a significance level (P < 0.05). Interestingly, DNA concentration was not significantly associated (P > 0.05) with pass/fail status; genotyping was successful for approximately 89% of low concentration (<50 ng/μl) samples and for 92% of DNA samples with a concentration ≥50 ng/μl.

The joint effects of DNA purity, turbidity, and saliva sample volume on genotyping outcomes were investigated. In a logistic regression model with sample pass/fail status as the dependent variable and saliva volume, DNA purity and DNA turbidity as predictive variables, each of the three variables had a significant effect on sample status. These results indicate that each variable exerts an independent effect on the probability of genotyping success. While saliva sample volume remained the variable with the largest effect (P = 6.8x10^-27^), DNA turbidity and purity where highly significant at P = 2.1×10^-7^ and P = 1.8×10^-4^, respectively. The odds of a sample being successfully genotyped for 95% of markers was higher when the saliva sample volume was >0.5 mL (OR = 9.3, 95% CI = [6.1, 14.8]) than when the volume was 0.5 mL and when the DNA purity was 1.8-2.0 versus otherwise (<1.8, OR = 2.4, 95% CI = [1.6, 3.7]). Increasing levels of Turbidity (A_320_, natural log transformed) reduced the odds of a sample being successfully genotyped.

There were significant associations between turbidity and DNA purity category (P < 10^-12^) and between turbidity and sample volume category (P < 10^-6^), but not between saliva sample volume category and DNA purity category. The relationship between turbidity and purity was as expected, with low purity associated with higher levels of turbidity. Interestingly, DNA from low volume saliva samples (i.e., 0.5 mL) had lower levels of turbidity than DNA from larger sample volumes, an observation consistent with earlier reports [9].

## Discussion

Incorporation of human biological sample collection for the purpose of genetics research has enhanced our understanding of biomarkers associated with disease and drug response [10-12] and has become routine in global clinical trials. While collection of a blood sample is the preferred sampling technique in many circumstances, alternative non-invasive saliva sampling is appropriate in certain cases and may have the advantage of being more acceptable to study participants, particularly in studies where no other invasive samples are collected. Concerns about reduced yield and quality of saliva DNA and the presence of contaminants appear unfounded with saliva DNA reported to be comparable to blood DNA when utilized on a variety of genotyping platforms [5].

The Oragene® OG-300 non-invasive saliva sampling kit requires study participants to collect their own saliva sample and in a global trial setting involving multiple investigator sites it may not be possible to observe collection practices and ensure that manufacturer recommended procedures are fully observed across all participants. Indeed, in the 13 global trials and 1468 subjects reviewed, 31.1% of study participants provided only 0.5 mL of saliva rather than the manufacturer recommended 2 mL. Interestingly, this rate varied among the studies, from 3% to 67%, indicating inconsistent collection practices. Whether this inconsistency could be addressed through improved training and information being made available to clinical site staff and study participants should be considered. It can take study participants up to thirty minutes to collect the recommended volume of saliva. A variety of conditions have been linked with xerostomia (dry mouth), as have certain medications [8,13] and increasing age, any of which may make it difficult to produce sufficient saliva. This study did not aim to evaluate subject demographics and genotyping success. However, based on the information available there was evidence of a borderline association between age and saliva volume, which corroborates other reports [8], and may warrant further investigation. Clearly, before utilizing saliva sampling, the study population should be considered.

Initial observations suggested that while the majority of saliva DNA samples performed similarly to blood DNA samples, a greater proportion of saliva DNA samples were likely to fail genotyping QC standards and that samples where the initial volume of saliva collection was 0.5 mL were significantly more likely to fail. Thus, we further evaluated the impact of saliva sample volume in 1468 subjects on DNA sample genotyping pass rate for 220 SNPs. Irrespective of the volume of saliva collection, the majority of DNA samples from saliva passed genotyping QC standards, returning genotype data for at least 95% of the SNPs tested, consistent with previous reports [6]. However, the sample pass rate for DNA derived from 0.5 mL saliva samples was significantly lower than that for DNA derived from saliva samples of larger volumes. In these clinical studies, saliva samples were sent to a central laboratory for pre-analytical processing. The sample volumes used for the current study were those reported by the central laboratory. The lowest reported volume was 0.5 mL, but it is possible that samples with <0.5 mL of saliva may have been included in the 0.5 mL group. Precise measures of volumes less than 0.5 mL could not be accounted for in this analysis and this is recognized as a limitation. Nonetheless it is clear from our data that low saliva sample volume was an important factor that influenced downstream genotyping outcomes. Hu et al. recently reported data in which DNA was extracted from 0.5 mL Oragene®/saliva mixture and that 90% of these samples successfully genotyped. In contrast approximately 80% of the 0.5 mL saliva DNA samples in this study had a successful genotyping outcome. This apparent discrepancy could be explained by the initial volume of saliva collection. Hu et al. did not report the volume of saliva collected from each study participant [6]. One possible explanation for our observations may be that insufficient volume of saliva collection results in an inappropriate ratio between saliva and Oragene® reagent which subsequently impacts downstream genotyping experiments.

Analysis by study demonstrated that for eight of the 13 clinical studies, the sample pass rate among the >0.5 mL saliva samples was statistically greater than that for 0.5 mL samples. For 3 studies the sample pass rate was numerically, but not statistically, higher in the >0.5 mL samples. Surprisingly, in 2 of the studies the sample pass rate was slightly higher in the 0.5 mL group compared to the >0.5 mL group (Figure 3). It was not possible to determine the reasons for the variability between studies, although factors such as whether study participants ate or drank prior to providing a sample may have contributed. A recent report suggested that more than 60% of DNA extracted from saliva samples may be bacterial or fungal in origin and that the percentage of amplifiable human DNA influences genotyping call rate [6]. It is possible that the 0.5 mL saliva samples analyzed in this study contained a significant percentage of non-human DNA and were more likely to fail genotyping due to insufficient concentrations of human DNA. The percentage of amplifiable human DNA was not determined in this study and this possibility cannot be excluded. However, it unlikely to fully explain our observations given that >80% of 0.5 mL saliva samples tested in this study provided robust genotype data. Further all but one of the clinical studies evaluated here were respiratory studies and many participants would have been familiar with providing sputum samples. It is possible that in some cases participants may have provided sputum rather than saliva which may have a downstream impact on DNA purification.

We evaluated whether DNA purity, contamination, turbidity, volume, concentration, and yield contributed to the likelihood of a successful genotyping outcome. Saliva volume, DNA purity and turbidity were significantly associated with a sample’s genotyping success or failure and together accounted for 18.6% of the variance in sample success. A multivariate logistic regression model indicated that these parameters exert independent effects. While 55% of samples had an A_260_/A_280_ ratio within the ideal value of 1.8-2.0, approximately 44% had values less than 1.8, indicative of more protein contamination. However, protein contamination was no more prevalent in DNA derived from 0.5 mL saliva samples compared to samples with higher volumes. Abraham et al. reported more protein contamination in saliva DNA samples compared to blood DNA and that saliva DNA was often more fragmented, although their study only compared 10 samples in each group [5]. DNA fragmentation was not considered in this study. In this study, DNA concentration was not significantly associated with pass/fail status, an observation consistent with an earlier report which suggested that increasing the absolute amount of DNA would not significantly improve genotyping success [6].

Based on our results, DNA purity and turbidity each play a role in determining whether a saliva DNA sample will pass QC criteria. However, neither parameter can be invoked to explain the even stronger role of low saliva sample volume. Additional factors must contribute, but the information available within this study was insufficient to establish a specific cause for the poorer performance of low volume saliva samples. In addition to the lack of more detailed information, another limitation of this study is that only one genotyping platform was evaluated. However, it seems likely these results would translate to other platforms, as is reported by others [5,6].

## Conclusions

Non-invasive saliva sampling provides an alternative where blood sampling is not desirable. The majority of saliva DNA samples provide high quality genotype data. However, collection of 0.5 mL volumes of saliva contributed to DNA samples being significantly less likely to pass genotyping QC standards and could not be explained by additional variables that may influence DNA sample genotyping pass rate. Thus, it is important to encourage all study participants to provide sufficient saliva sample to minimize potential loss of data in downstream genotyping experiments.

## Competing interests

The authors declare that they have no competing interests. DJP, MM, JDB, KWJ and AJN are employees of and hold stock in GlaxoSmithKline.

## Authors’ contributions

JDB, KWJ and AJN contributed to the experimental design. DJP, MM, JDB, KWJ and AJN contributed to data review, interpretation and manuscript preparation. MM contributed to the design of the statistical analysis plan and performed the statistical analysis. DJP drafted the manuscript. All authors approved the final manuscript.

## Pre-publication history

The pre-publication history for this paper can be accessed here:

http://www.biomedcentral.com/1755-8794/6/20/prepub

## Supplementary Material

Additional file 1 Institutional Review Board/Independent Ethics Committees that approved genetics research described in “Saliva sampling in global clinical studies: the impact of low sampling volume on performance of DNA in downstream genotyping experiments”. Click here for file

Additional file 2 Concordance rate for SNP genotype data for blood and saliva derived DNA. Click here for file
