# Supplementary material for: Saliva sampling in global clinical studies: the impact of low sampling volume on performance of DNA in downstream genotyping experiments
Source: BMC Med Genomics. 2013 Jun 10;6:20. doi: 10.1186/1755-8794-6-20 (PMC3698156; doi:10.1186/1755-8794-6-20)
Supplement: Additional file 1 — Institutional Review Board/Independent Ethics Committees that approved genetics research described in “Saliva sampling in global clinical studies: the impact of low sampling volume on performance of DNA in downstream genotyping experiments”. [file 1755-8794-6-20-S1.pdf]

Additional Materials: Institutional Review Board/Independent Ethics Committees that approved genetics research described in "Saliva sampling in global clinical studies: The impact of low sampling volume on performance of DNA in downstream genotyping experiments*"

Coast IRB, LLC 901 Calle Amanecer Suite 260 San Clemente, CA 92673 USA
Comité de Docencia e Investigación de la Clinica del Torax.
Comité de Docencia e Investigación de la Fundacion CIDEA
Comité de Docencia e Investigación del Centro de Alergia e Inmunologia
Comité de Docencia e Investigación del Centro INSARES
Comité de Docencia e Investigación del Centro Investigaciones Patologicas Respiratorias
Comité de Docencia e Investigación del Centro VITAE
Comité de Docencia e Investigación del Hospital
Comité de Docencia e Investigación del Hospital A.A Cetrangolo
Comité de Docencia e Investigación del Hospital Ramos Mejia
Comité de Docencia e Investigación del Instituto del Hospital Aeronautico Central
Comité de Docencia e Investigación del Instituto Diagnostico Medico Bioquimico
Comité de Docencia e Investigación del Sanatorio San Jose
Comité Revisional e Institucional AERI
Compass IRB, 5416 East Baseline Road, Suite 120, Mesa, AZ 85206
Copernicus Group IRB 1 Triangle Drive, Suite 100 P.O. Box 110605 RTP, NC 27709
Coriell Institute for Medical Research IRB, Control Samples acquired from NIGMS Human Genetic Cell Repository
Copernicus Group IRB, 118 MacKenan Drive, Cary, NC 27511
CPP Sud Méditerranée III Faculté de médecine Avenue Kennedy CS 8302130908 NIMES Cedex 2
Ethik-Kommission der Landesärztekammer Hessen Im Vogelsgesang 3 60488 Frankfurt am Main Germany
Ethik-Kommission des Landes Sachsen-Anhalt c/o Landesamt für Verbaucherschutz Kühnauer Str. 70 06846 Dessau Germany
Great lakes IRB, 216 N Second Street, PO Box 182, Peotone, IL 60468
Harrison Institutional Review Board 16B South Main Street London, OH 43140
Health Sciences IRB, University of Wisconsin, Madison, VA Hospital, RM B3088, 2500 Overlook Terrace, Madison, WI 53705
Institutional Review Board, Faculty of Medicine Ramathibodi Hospital, Mahidol University, 270 Rama 6 Road, Bangkok 10400 Thailand
Khon Kaen University Ethics Committee for Human Research 123 Mitraphap Road Dean's Office 6th Floor Faculty of Medicine Khon Kaen University, Khon Kaen 10400 Thailand
Komisja Bioetyczna Uniwersytetu Medycznego w Bialymstoku ul. Jana Kilinskiego 1 15 - 089 Bialystok Poland
lnstitutional Review Board Faculty of Medicine Chulalongkorn University 1873 Rama 4 Road Patumwan, Bangkok 10330 Thailand
Louisiana State University Health Science Center-Shreveport Institutional Review Board, 1501 Kings Highway, Shreveport, LA 71103
Mary Mediatrix Medical Center
METC Postbus 1350 5602 ZA Eindhoven
National Jewish Medical and Research Center Institutional Review Board 1400 Jackson Street Denver, CO 80206
Optimum ERB 231 King Street East Oshawa, Ontario L1H 1C5
Partners Human Research Committee, 116 Huntington Avenue Suite 1002 Boston, MA 02116
Pharma Ethics Committee, 123 Amcor Road, Lyttelton Manor, 0157
Regional Komitè for medisinsk og helsefaglig forskningsetikk, Helseregion Midt-Norge, Medisinsk teknisk forskningssenter, 7489 Trondheim Norway
Research and Ethics Committee of Veterans Memorial Medical Center

*Individual Institutional Review Board/Independent Ethics Committees may have approved genetics research in the clinical studies at more than one clinical site.

Additional Materials: Institutional Review Board/Independent Ethics Committees that approved genetics research described in "Saliva sampling in global clinical studies: The impact of low sampling volume on performance of DNA in downstream genotyping experiments*"

Research Ethics Committee Faculty of Medicine, Siriraj Hospital, Mahidol University, 2 Prannok Road, Adulyadejvikrom Building, 10th floor, Bangkoknoi, Bangkok 10700 Thailand
Saint Joseph Mercy Health System Clinical Research Committee 5301 East Huron River Drive P.O. Box 995 Ann Arbor, MI 48106-0995
Schulmann Associates IRB Inc., 4290 Glendale Milford Road, Cincinnati, OH 45242
Southampton and South West Hampshire REC A, First Floor, Regents Park Surgery , Park Street , Shirley , Southampton, SO16 4RJ
St. Francis Hospital & Medical Center IRB 114 Woodland Street Hartford, CT 06105
Sterling IRB 6300 Powers Ferry Road Suite 600-351, Atlanta, GA 30339
STL De La Salle Medical Center
The Ethical Committee on Research Involving Human Subjects, Faculty of Medicine, Siriraj Hospital, Mahidol University, 2 Prannok Road, Aduiyadejvikrom Building 10th floor, Bangkoknoi, Bangkok 10700 Thailand
The IRB/EB at the Philippine General Hospital, University of the Philippines, Metro Manila, Philippines
The University of Chicago Institutional Review Board Office of Research Services Section of Regulatory Compliance McGiffert Hall, 2nd Floor 5751 S. Woodlawn Ave. Chicago, IL 60637
The University of Chicago IRB, 5841 S. Maryland Avenue, AMB-S138 MC 1108 Chicago, IL 60637
UCLA Office for Protection of Research Subjects 11000 Kinross Avenue, Suite 102 Box 951694 Los Angeles, CA 90095
University Hospitals of Cleveland Research Institute 11100 Euclide Avenue Lakeside Bldg., Room 1400 Cleveland, OH 44106
University of Cape Town, Research Ethics Committee, Faculty of Health Sciences, Room E53-24, Groote Schuur Hospital, Old Main Building, Observatory, 7925
University of South Florida IRB, 12901 Bruce B. Downs Blvd. MDC 035, Tampa, FL 33612-4799
University of Stellenbosch Committee for Clinical Trials, Faculty of Health Sciences, Suite 5006, 5th Floor, Fransie van Zijl Drive, Parow, 7500
Wake Forest University Health Board, The Office of Research and Development, Medical Center Blvd. Winston-Salem, NC 27157
Wake Forest University Health Sciences IRB, School of Medicine, Bowman Gray Campus, Medical Center Blvd. Winston-Salem, NC 27157-1023.
Washington University School of Medicine 660 S. Euclid Ave. St. Louis, MO 63110
Wayne State University Human Investigation Committee, 4201 St. Antoine Blvd. UHC-8G, Detroit, MI 48201
Western IRB 3535 Seventh Avenue, SW Olympia, WA 98502-5010

*Individual Institutional Review Board/Independent Ethics Committees may have approved genetics research in the clinical studies at more than one clinical site.
