# Supplementary material for: Saliva sampling in global clinical studies: the impact of low sampling volume on performance of DNA in downstream genotyping experiments
Source: BMC Med Genomics. 2013 Jun 10;6:20. doi: 10.1186/1755-8794-6-20 (PMC3698156; doi:10.1186/1755-8794-6-20)
Supplement: Additional file 2 — Concordance rate for SNP genotype data for blood and saliva derived DNA. [file 1755-8794-6-20-S2.pdf]

Supplementary information

Table 1 Concordance rate for SNP genotype data for blood and saliva derived DNA

	#Comparisons	#Concordant	#Discordant	Concordance Rate
Blood-Derived DNA	14697	14634	63	99.57%
Saliva-Derived DNA	20994	20927	67	99.68%

Genotyping of 551 saliva and 352 blood derived DNA samples were genotyped on the OmniQuad BeadChip and FRET-KASPar platforms. Of the 77 single nucleotide polymorphisms (SNPs) tested on by KASPar assay, 43 of these were also tested on the Illumina Human OmniQuad BeadChip allowing concordance comparisons between platforms for blood and saliva derived DNA. Comparison of genotyping results between the OmniQuad BeadChip and KASPar assays demonstrated a >99.5% concordance rate for both blood and saliva derived DNA.
